# PET/CT is a cost-effective tool against cancer: synergy supersedes singularity

**DOI:** 10.1007/s00259-016-3414-5

**Published:** 2016-05-13

**Authors:** Barbara Malene Fischer, Barry A. Siegel, Wolfgang A. Weber, Konrade von Bremen, Thomas Beyer, Antonis Kalemis

**Affiliations:** 1 Department of Clinical Physiology, Nuclear Medicine and PET, Rigshospitalet, University of Copenhagen, Copenhagen, Denmark; 2 Division of Nuclear Medicine, Mallinckrodt Institute of Radiology and Alvin J. Siteman Cancer Center, Washington University School of Medicine, Saint Louis, MO USA; 3 Department of Radiology, Memorial Sloan Kettering Cancer Center, New York, NY USA; 4 SWAN Isotopen AG, c/o Inselspital, 3010 Bern, Switzerland; 5 European Industrial Association for Nuclear Medicine and Molecular Healthcare (AIPES eeig), Brussels, Belgium; 6 Center for Medical Physics and Biomedical Engineering, General Hospital Vienna, Medical University of Vienna, Waehringer Guertel 18-20/4 L, 1090 Vienna, Austria; 7 Philips, Advanced Molecular Imaging, Guildford, GU2 8XG UK

Since its introduction in 2000, PET/CT has become a widespread and effective imaging tool for the diagnosis and management of patients with cancer. Today, over 5,000 combined PET/CT systems are in clinical operation worldwide. As a core component of PET/CT, PET is a highly sensitive imaging technique capable of detecting as few as one million cancer cells [1]. Its inherent quantitative nature enables accurate, reproducible measurements of radiopharmaceutical uptake in the tumour during diagnostic work-up, therapy and treatment follow-up. Technological advances in PET imaging technology go hand in hand with the development of highly specific PET probes, with most of them only now being available for on-site use.

Combined PET/CT has been shown to have exceeded the expectations laid out in a seminal paper by Wahl et al. [2], who referred to combined imaging methods as “anatometabolic” imaging, thus pointing to the inherent potential benefits of combining complementary imaging methods. Numerous studies have demonstrated the increased diagnostic accuracy of PET/CT compared with that of either PET or CT alone. As a result, PET/CT has become one of the most effective imaging modalities in oncology. Clinical users and manufacturers have also started to adopt standardized acquisition and reporting protocols as a prerequisite for the adoption of any imaging modality in guidelines for patient management.

Furthermore, PET/CT has shown significant promise for reducing the cost of cancer management by improving the accuracy of both diagnosis and staging, thereby helping to avoid expensive, futile treatments (such as curative intent surgery and radiation therapy in patients demonstrated by PET to have advanced disease) and associated side effects. Overall, PET/CT has the potential to increase a patient’s quality-adjusted life years (QALYs) and reduce the cost burden over time to the healthcare system by identifying the most appropriate treatment. However, the potential of PET as a tool to help in the management of cancer patients has not yet been reflected in the extent of its adoption. Here, we attempt to summarize the main reasons for this observation.

## Clinical evidence

One of the most widely adopted application of PET/CT is for the preoperative staging of non-small-cell lung cancer (NSCLC) using [^18^F]fluorodeoxyglucose (FDG). Randomized clinical trials have established the the value of PET/CT over conventional CT for the staging of NSCLC [3, 4]. More recent studies have analysed the cost-effectiveness of PET/CT for preoperative staging of NSCLC and have demonstrated that the clinical use of PET/CT for staging of the disease leads to significant decreases in surgery and radiotherapy rates and an increase in chemotherapy use [5]. Cost-effectiveness analyses performed alongside randomized clinical trials have established that PET/CT provides accurate preoperative staging of NSCLC and its use leads to cost savings. The systematic use of PET/CT has been shown to decrease the number of futile thoracotomies and lower the costs associated with lung cancer diagnosis and treatment [6, 7]. NSCLC patients receiving the most appropriate treatment have better quality of life. Similar results from a 2-year multicentre study on head and neck squamous cell carcinoma follow-up have recently been published. Patients receiving PET/CT surveillance had equal survival probability, while unnecessary surgery and potential complications were avoided, and its use saved £1,492 per patient for the duration of the study [8].

PET/CT imaging is also emerging as a powerful tool for identifying and localizing primary and recurrent prostate cancer. The use of PET/CT in prostate cancer has benefited from the development of promising radiotracers, including [^11^C]choline, [^18^F]fluorocholine, [^11^C]acetate, [^18^F]FACBC and, recently, agents targeting prostate-specific membrane antigen (PSMA) [9–11]. [^11^C]Choline- and [^18^F]fluorocholine PET/CT are being increasingly used in the US, Europe and Japan to detect locally recurrent and metastatic prostate cancer. Early clinical results suggest that the novel PSMA-targeted radiotracers may prove even more effective [12]. Since prostate cancer is a biologically and clinically heterogeneous disease, ranging from indolent to aggressive forms, PET/CT may be critical as well as cost-effective in therapy selection and management of prostate cancer.

Hypoxic tumours are associated with an aggressive phenotype, therapy resistance, increased risk of metastasis, and an overall poor prognosis [13]. PET/CT is the preferred method for identifying tumour hypoxia due largely to its high specificity and sensitivity, and quantification capabilities, so an increasing number of hypoxia PET tracers are being evaluated in the clinic. For instance, studies have shown promising results with [^18^F]fluoromisonidazole (FMISO) and [^18^F]fluoroazomycin-arabinofuranoside (FAZA) for identifying hypoxia in a variety of tumour types, including gliomas, lung, and head and neck tumours [14]. Thus, in the future PET/CT may help guide treatment decisions by identifying patients who are likely to benefit from hypoxia-targeted therapy. Moreover, there is also an interest in using PET/CT for radiotherapy treatment planning because of its ability to identify and delineate hypoxic areas that require a higher dose [15].

The few clinical examples discussed above show how PET/CT can help select the appropriate cancer treatment, so that ineffective therapies can be avoided or quickly discontinued reducing both cost and emotional burden on the patient. PET/CT can also be used to evaluate the effectiveness of new chemotherapeutic agents. The costs of cancer drug development continue to rise at an unsustainable rate and a key driver of this escalation is the failure of experimental drugs late in the development process [16]. PET/CT can be used to investigate the pharmacodynamics of new drugs and to identify eventual drug failure at earlier stages of development. This may ultimately help to contain the costs of drug development. Clinical trials of ineffective drugs can be stopped in a timely fashion, an important consideration in view of the limited funds available. Resources can be redirected to those drugs that are validated as effective throughout all stages of drug development. For example, iniparib was reported to be a poly(ADP-ribose) polymerase (PARP) inhibitor with promising results for treating patients with triple-negative breast cancer. However, recent studies have suggested that it is not a PARP inhibitor at clinically relevant doses in patients [17]. This resulted in the waste of US$285 million, which could have been avoided, or greatly reduced with the use of appropriate preclinical PET studies.

## Barriers to clinical adoption

Despite the documented clinical benefits of using PET/CT for oncology applications, there are several significant barriers to its wider clinical adoption:

1. Clinicians referring patients for nuclear medicine examinations need regular training opportunities to better value the diagnostic information that PET/CT images can provide. The lack of anatomical resolution in PET-only images hindered fast clinical adoption of this technology among traditional, radiology-driven medical experts in the early period of PET development. The introduction of integrated PET/CT systems has largely overcome this barrier, but continued education of referring physicians is still essential.
2. Regulation of the production of radiopharmaceuticals varies across the world. To date, radiopharmaceuticals are considered as full pharmaceuticals, mandating correspondingly complex handling, even though any effect of the active pharmaceutical component of a radiopharmaceutical is well below therapeutic levels. The increasingly demanding regulatory requirements are a major concern for the generally small radiopharmaceutical companies. Innovative new radiopharmaceuticals are difficult to bring to the clinic and the market. As a consequence, only few industrial partners set out on this steep path.
3. PET/CT coverage policies of government agencies and private payers are both variable and restrictive. While coverage has been expanded in the US, it varies considerably within the European Union, with Germany and Austria promoting essentially a “nil reimbursement” policy for public healthcare. Large randomized clinical trials, requested by various national health authorities, are prohibitively expensive in most countries.

## Recommendations to overcome barriers to wider adoption of PET/CT

Regulatory agencies that determine coverage policies require peer-reviewed scientific evidence to show that a new radiopharmaceutical or technology leads to improvements in patient management and health outcomes. In the field of oncology, such evidence is generally required on a cancer-specific and indication-specific basis. This often creates a “catch 22” scenario in which coverage cannot be attained without evidence, but there is no viable way to pay for the PET/CT scans to acquire the evidence without coverage. The experience in the US may serve as an example of how to overcome the coverage barrier. In the US, the Centers for Medicare & Medicaid Services (CMS) administer the federal health insurance programs and CMS policies dominate the marketplace. In 2004, CMS proposed expansion of the coverage of oncological PET based on a new policy called “coverage with evidence development” (CED) that allowed coverage of promising drugs, biologicals, devices, diagnostics and procedures in order to evaluate their clinical benefit for a limited time. As a result, the National Oncologic PET Registry (NOPR) was formed to assess the effect of PET on referring physicians’ *intended* patient management across a wide spectrum of cancer indications for PET not then covered by CMS.

NOPR is funded by charging the PET facilities a fee of US$50 for each patient placed on the registry. Participating clinicians complete a pre-PET and a post-PET questionnaire to determine whether the imaging changed their intended patient management. The data that NOPR collected from 2006 to 2013 demonstrated that PET changed the intended management in about 40 % of patients overall [18]. When used for monitoring chemotherapy, the treatment was altered in about 78 % of patients when PET indicated that the patients’ disease was getting worse [19]. Based on the NOPR results and the expanding literature on the efficacy of PET, CMS expanded PET coverage for diagnosis and initial staging for most cancers in 2009 and further expanded coverage for other indications, such as treatment monitoring, restaging and detection of suspected recurrence, in 2013 [20].

The NOPR observational studies reflect “real-world” clinical practice with modern PET/CT systems and are based on large patient cohorts. However, these studies lack control groups and look at the change in *intended* patient management based on PET results rather than *actual* patient management. The new Imaging Dementia - Evidence for Amyloid Scanning (IDEAS) study, another CMS coverage with evidence development study in the US, will take a more rigorous approach by evaluating whether brain amyloid PET findings lead to a changes in actual patient management and improvements in outcomes. It will use Medicare claims to identify concurrent propensity-matched controls who have not had amyloid PET imaging for comparison with the study participants. Another innovative aspect of the IDEAS study is that the three industrial providers of amyloid radiopharmaceuticals are collaborating to finance the trial. Both NOPR and IDEAS have the merit of overcoming coverage barriers by revising the way imaging technologies are clinically evaluated.

For evaluation of PET radiopharmaceuticals, the rigid obligation to use randomized controlled trials is expensive, not always effective, and sometimes not even feasible [21]. When evaluating the clinical benefit of PET/CT, it is important first to distinguish between direct and indirect clinical benefits [21]. If PET/CT can replace an alternative invasive procedure, such as surgical staging, then it has the direct clinical benefit of avoiding side effects from the invasive procedure. In such cases, noninvasive PET/CT could be performed in addition to the standard invasive procedure in each patient, in order to confirm that its diagnostic accuracy is as good as that of the standard. Accuracy studies are well-suited to showing the impact on patient management; there is no need to consider long-term health outcomes with a randomized clinical trial. If instead PET/CT replaces another imaging modality, it has no direct benefit. It has an indirect benefit if it provides both improved diagnostic accuracy and changes in patient management that lead to improved health outcomes. When assessing PET/CT for an indirect clinical benefit, decision modelling should be used initially. If decision modelling is inconclusive, then randomized clinical trials should be initiated that focus from the outset on whether PET/CT improves health outcomes such as quality of life.

Finally, continuous training efforts involving all stakeholders in the decision-making process and actual referral and acquisition procedures should take place. Such training can benefit from adapting successful national or societal educational concepts from other countries. Here, the scientific associations, such as the European Society of Radiology and the European Association of Nuclear Medicine can provide organizational support and governance, as laid out several years ago in a joint white paper [22, 23].

Any change in patient management that leads to the avoidance or discontinuation of an ineffective therapy can be considered as a “surrogate” for an improved health outcome. CED has the potential to become the standard approach for evaluating new radiopharmaceuticals and technologies to facilitate the collection of clinically valuable data. In this way, PET/CT can be made widely available for proven oncology indications based on appropriate evidence of its clinical value and cost-effectiveness.
